# P-702. Epidemiology of Respiratory Pathogens and the Impact of Seasonality, Patient Demographics, and Diagnostic Testing Approach: A Retrospective Analysis from 2022 to 2023

**DOI:** 10.1093/ofid/ofae631.898

**Published:** 2025-01-29

**Authors:** Kaisha Gonzalez

**Affiliations:** Diasorin, Laurel, Maryland

## Abstract

**Background:**

Respiratory tract infections (RTIs) are a significant concern for public health due to their widespread transmission and high rates of illness and death reported globally. The use of multiplex molecular methods has allowed for the detection of a broad range of respiratory viruses and their subtypes, as well as some bacteria. This study investigates the epidemiology of respiratory pathogens detected in different demographic populations, emphasizing the importance of a flexible diagnostic testing approach to adapt to the complex dynamics of respiratory pathogens during the peak season.

Distribution Rates of Respiratory Targets in Young, Adult, and Elderly Patients (n=1,520)

**Figure:**
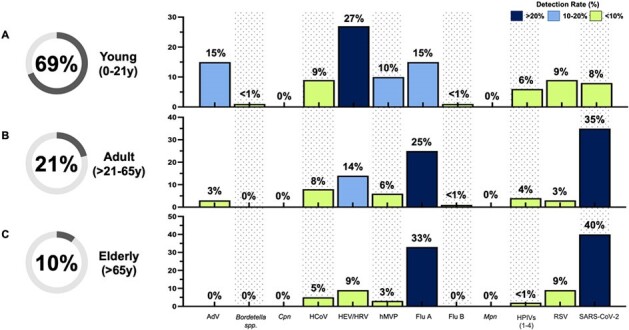

Analysis of distribution rates of 19 respiratory targets during the respiratory season of 2022-2023, stratified by age.

**Methods:**

During the 2022-23 respiratory disease season, the Diasorin LIAISON PLEX^®^ Respiratory *Flex* Assay was used to prospectively test 1,520 patients across six clinical sites in the United States. As part of an observational study, retrospective analyses were conducted to compare the detection rates and frequencies of 19 respiratory targets (14 viral and 5 bacterial targets). The results were stratified by age.

**Results:**

Many respiratory pathogens displayed age- and season-specific patterns. Enterovirus/rhinovirus was detected more frequently in patients < 21 years old (27%) than in the adult group (14%) and elderly group (9%), p< 0.01. Flu A prevalence was found to be higher in adults (21-65 y) at 25% and 33% in the elderly ( >65 y) population, p≤0.05. Individuals < 21 years had a lower incidence of SARS-CoV-2 (8%) compared to adults (35%) and the elderly (40%), p< 0.001. The most common viruses found in all three populations were flu A, human coronavirus, SARS-CoV-2, and flu A-H1, while the other pathogens were more population-specific.

**Conclusion:**

The results of the study offer valuable insights into the complex dynamics of respiratory pathogens. They emphasize the importance of flexible multiplex PCR testing strategies that consider the different circulating patterns of these pathogens across different populations. This approach can help customize respiratory testing based on factors such as seasonality, patient demographics, and acceptable costs, and in line with diagnostic stewardship, adapt testing protocols to meet the specific needs of respiratory testing.

**Disclosures:**

**All Authors**: No reported disclosures

